# BanglaEcomReviewCorpus: A dataset for e-commerce product review sentiment analysis

**DOI:** 10.1016/j.dib.2026.112663

**Published:** 2026-03-08

**Authors:** Umme Ayman, Md. Tanvir Ahmed Akash, Taslima Akhter, Saiham Zaman Mridul, Yadab Sutradhar

**Affiliations:** a Department of Computer Science and Engineering. Daffodil International University, Bangladesh; b Department of Information and Communication Technology, Comilla University, Bangladesh

**Keywords:** Bangla text classification, Bangla language, Text classification, Sentiment analysis, Natural language processing

## Abstract

Online shopping has become an integral part of modern life, connecting consumers to a wide array of products and services. Customer feedback plays a crucial role in shaping business strategies, enhancing service quality, and driving product innovation, making it essential for understanding consumer behaviour and preferences. For analysing those feedbacks, a dataset is Collected from popular websites such as Daraz, Bikroy.com, Picabbo, Shajgoj, and others, this dataset comprises 8685 labeled items reflecting diverse customer feedback. Sentiment categories include 3012 positive, 2881 negative, and 2792 neutral sentences, offering balanced representation for fair sentiment analysis. This dataset is ideal for natural language processing (NLP) tasks, enabling advanced sentiment analysis and exploration of consumer behaviour. It incorporates a range of statistical studies, including summary statistics, histograms, and linguistic patterns such as unigrams, bigrams, and trigrams. Additionally, visualizations like word clouds provide insights into the dataset's structure and linguistic diversity. To ensure data integrity, rigorous collection methods, anonymization, and preprocessing techniques were employed. This publicly available dataset serves as a valuable resource for advancing sentiment analysis, improving business strategies, and supporting interdisciplinary research. It enables insights into customer behavior, aids product and service development, and can be used in both academic teaching and AI training. By capturing feedback from diverse e-commerce platforms, the dataset fosters collaboration across fields such as sociology, linguistics, and psychology.

Specifications Table

**Table:** 

Subject	Computer Sciences
Specific subject area	Sentiment Analysis, Public Sentiment, Natural Language Processing, Data Science, Machine Learning, Bengali Text Classification
Type of data	Text Files (xlsx-formatted)
Data collection	The dataset was meticulously curated to capture the wide spectrum of customer sentiments surrounding online shopping experiences across various platforms. Data sources included customer reviews from popular websites such as Daraz, Bikroy.com, Picabbo and others. The dataset contains 8685 labeled items, categorized into 3012 (34.7 %) positive, 2881 (33.2 %)1 negative, and 2792 (32.1 %) neutral sentiments, achieving a balanced distribution while reflecting the natural diversity of real-world feedback. To ensure quality and reliability, a multi-step validation process was employed. This dataset is an invaluable resource for analysing sentiment dynamics, exploring consumer behaviour, and creating advanced natural language processing applications in the e-commerce sector.
Data source location	Publicly open Bangladeshi online shopping platforms: Daraz, Bikroy.com, Picabbo and others, and do not correspond to a specific location.
Data accessibility	Repository name: Mendeley Data Data identification number: 10.17632/kkzfrvhbhp.2 Direct URL to data: https://data.mendeley.com/datasets/kkzfrvhbhp/2https://data.mendeley.com/datasets/kkzfrvhbhp/1

## 1 Value of the Data

• This dataset provides a structured and manually annotated collection of Bangla-language customer reviews sourced from multiple public e-commerce platforms. It contributes to the growing need for high-quality text data in low-resource languages and supports the development of language-specific tools for sentiment analysis and opinion classification.
• The dataset facilitates the development of sentiment classifiers for computational applications capable of distinguishing between various emotional tones across multiple textual formats, such as reviews, social media posts, and forum discussions. This precision enhances the performance of NLP tools in market research, customer satisfaction analysis, and brand reputation management by improving their ability to interpret context-sensitive feedback.
• For businesses, marketers, and researchers studying customer behaviour, product satisfaction, and service quality, the dataset is an invaluable resource. Its balanced sentiment distribution offers comprehensive insights into the emotional landscape of online shopping experiences, enabling interdisciplinary studies into consumer psychology, decision-making, and digital consumerism.
• In an educational context, this dataset provides authentic, real-world content for teaching sentiment analysis, consumer behaviour studies, and data science techniques. It can be integrated into courses for data scientists, business analysts, and researchers, providing students with practical experience in using NLP to analyse and interpret customer feedback in the e-commerce sector.
• Finally, by offering a standardized method for analysing customer feedback across multiple online platforms, this dataset contributes to the advancement of NLP research in e-commerce. It sets a foundation for future datasets that explore customer sentiment in different languages and regions, encouraging inclusivity and expanding the scope of computational linguistics to better understand global consumer experiences.

## 2 Background

Customer dissatisfaction with service, product quality, and shopping experiences remains a common issue in e-commerce. With the rise of various online shopping platforms, diverse consumer feedback has become crucial for understanding public sentiment. Yet, this feedback remains underexplored in computational research, limiting the development of advanced sentiment analysis tools and opinion mining applications using natural language processing (NLP).

Most existing studies focus on global platforms like Amazon or eBay and major international brands. However, there is a lack of comprehensive, culturally relevant datasets from regional platforms, particularly in countries like Bangladesh. This gap restricts the creation of tailored NLP tools for local markets.

To address this, we present a curated dataset of 8685 Bangla-language customer reviews collected from platforms such as Daraz, Bikroy.com, Picabbo, and others. The dataset includes 3012 positive, 2881 negative, and 2792 neutral reviews, offering a balanced view of customer sentiment.This dataset supports sentiment analysis, opinion mining, and the development of context-aware NLP models for e-commerce. It enables applications like sentiment tracking, satisfaction analysis, and product feedback systems. The resource benefits NLP researchers, data scientists, and businesses by enhancing the understanding of customer behavior and supporting data-driven, customer-centric strategies.

## 3 Data Description

Online shopping has emerged as a cornerstone of modern commerce, providing consumers with unmatched convenience and access to diverse products and services. Customer feedback plays an essential role in refining service quality, informing business strategies, and fostering innovation, making it crucial to study consumer sentiment comprehensively. However, the lack of sentiment analysis tools tailored to consumer feedback in Bangla language spoken by over 272.8 million people poses challenges for businesses and researchers alike in understanding and addressing customer needs [1].

**Fig. 1:**
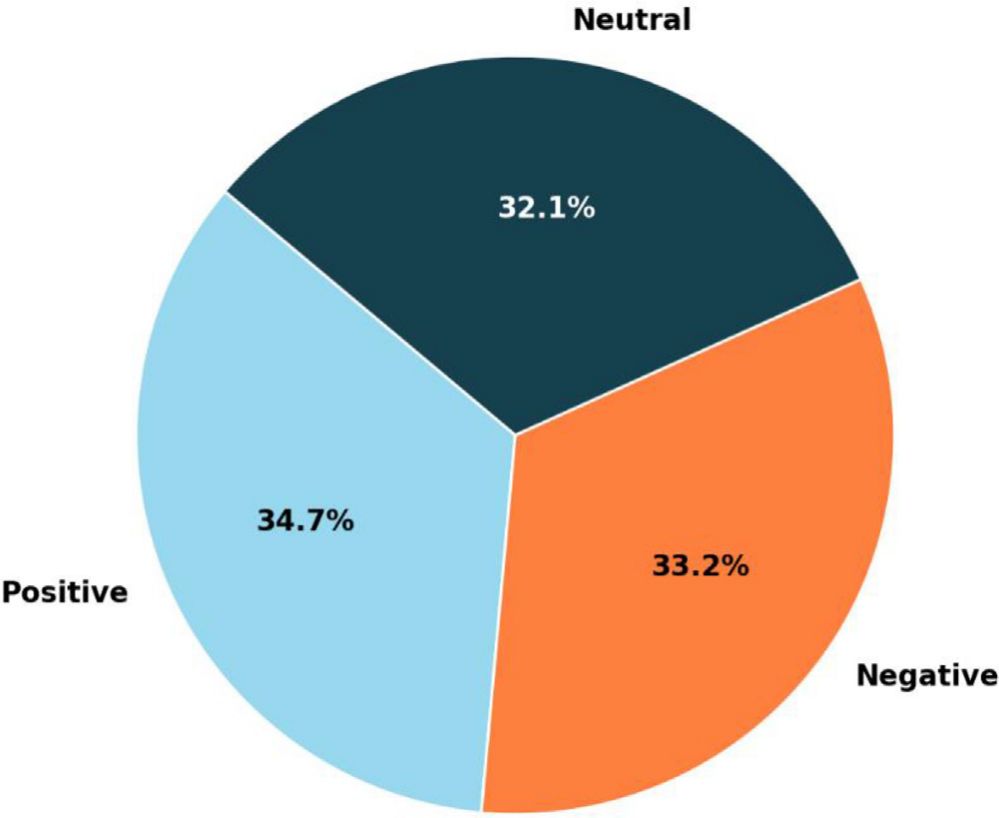
Class distribution of positive, negative and neutral levels of dataset.

To bridge this gap, we present a new dataset titled BanglaEcomReviewCorpus.xlsx, which includes 8685 labeled sentences capturing sentiment (positive, negative, and neutral) from popular online shopping platforms. This dataset represents real-world customer feedback and offers a critical tool for developing natural language processing (NLP) applications in Bengali, especially in the domain of e-commerce. The dataset has undergone extensive preprocessing to ensure integrity, including anonymization and validation for reliability and robustness. Statistical studies such as summary statistics, histograms, and linguistic patterns (unigrams, bigrams, trigrams) provide deep insights into the dataset’s structure and linguistic diversity. Visual tools, including word clouds, further enhance understanding of consumer feedback trends. This dataset is a vital resource for researchers exploring sentiment analysis [2], NLP applications, and consumer behaviour in Bengali. It offers opportunities for businesses to improve customer service, develop product insights, and refine marketing strategies. Additionally, it supports interdisciplinary studies in sociology, psychology, and linguistics, contributing to a deeper understanding of consumer decision-making and public opinion. The accompanying description provides an overview of the dataset structure, variables, and annotations to help researchers fully utilize its potential. Table 1 outlines the dataset's components, while Fig. 1 visualizes the sentiment class distribution: 3012 positive (34.7 %), 2881 negative (33.2 %), and 2792 neutral (32.1 %) items. This dataset marks a significant advancement in Bengali sentiment analysis resources and serves as a foundation for future research in the socio-linguistic aspects of e-commerce and customer engagement.

**Table 1:** Dataset description with attributes and possible values.

**Fig. 2:**
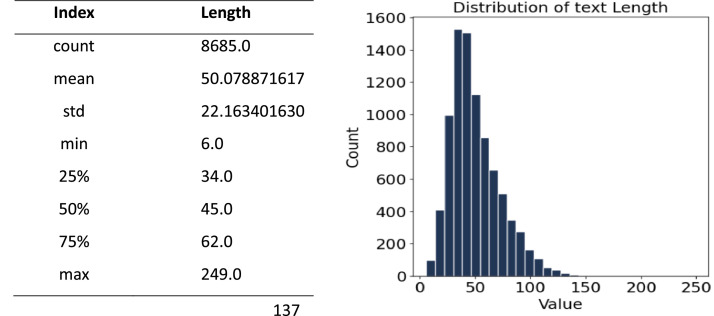
The frequency distribution of Bangla text length for the dataset.

Fig. 2 shows the overall distribution of text lengths in the BanglaEcomReviewCorpus, which includes 8685 sentences. The distribution is clearly right-skewed, meaning shorter sentences appear more frequently, while longer ones are relatively rare. Most sentences are between 30 and 70 characters long, with a median length of 45 characters. On average, sentences are about 50 characters long, with a standard deviation of 22, indicating a moderate level of variation in length. The shortest sentence in the dataset is just 6 characters, whereas the longest stretches to 249 characters, pointing to the presence of a few unusually long or highly detailed reviews.

Table 2 breaks down sentence length statistics based on sentiment categories—positive, negative, and neutral—within the BanglaEcomReviewCorpus. It provides insight into how users express their opinions differently depending on sentiment.There are 3012 sentences in the positive class. With a standard deviation of 19.57 and an average length of 43.89 characters, these sentences exhibit moderate diversity. The majority of suitable statements are between 32 and 51 characters, indicating that people typically provide consistent and brief positive feedback.There are 2881 sentences in the negative class, and they are often wider and more varied in length. The length is the greatest of the three groups, averaging 64.55 characters with a standard deviation of 23.35. The majority of negative sentences are between 48 and 79 characters long, indicating that when people are unsatisfied they frequently write deeper inspections.

**Table 2:** Statistical analysis for positive, negative, and neutral sentiments.

Sentiment	Count	Mean	Std	Min	25 %	50 %	75 %	Max
Positive	3012	43.894754	19.565911	7	32	40	51	143
Negative	2881	64.545644	23.351215	6	48	63	79	249
Neutral	2792	41.822350	15.073638	7	31	40	51	116

Overall, the BanglaEcomReviewCorpus dataset reveals a balanced proportion of sentences in all levels. The diversity in sentence lengths is comparable across levels, with the neutral category having the largest maximum sentence length. This thorough statistical summary aids in understanding the dataset's features, which are critical for constructing and verifying models for NLP applications in Bengali.

**Fig. 3:**
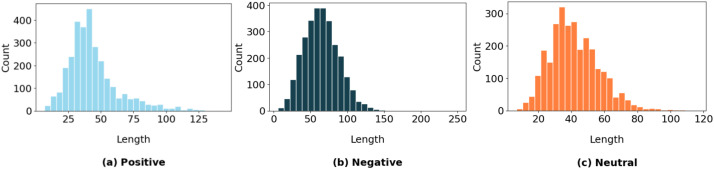
The frequency distribution of positive, negative, and neutral sentiments (a, b,c).

Fig. 3 compares the distribution of sentence lengths across the three sentiment categories: positive, negative, and neutral. The negative class has a wider and more spread-out distribution, indicating that these reviews often vary significantly in length, some being short, while others are much longer and more detailed. On the other hand, the positive and neutral classes have narrower, more concentrated distributions, suggesting that users generally write these types of reviews using more consistent and similar lengths. The visual clearly highlights how users tend to be more elaborate when sharing negative experiences, while keeping things simpler when writing positive or neutral feedback.

**Fig. 4:**
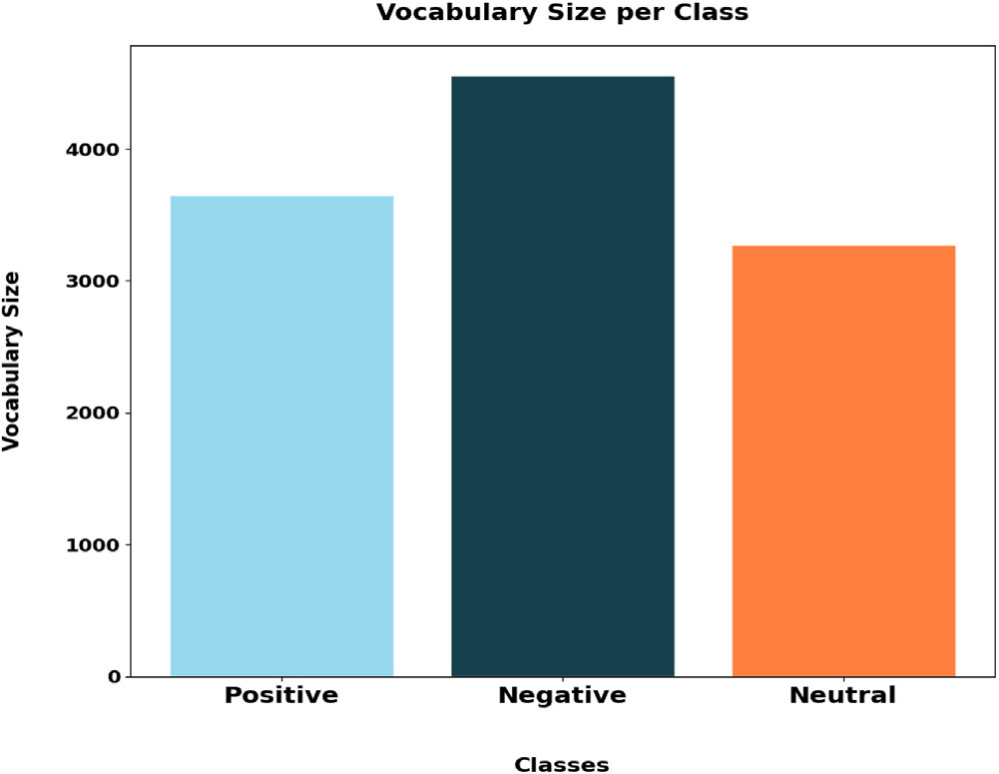
Vocabulary sizes per class.

Fig. 4 illustrates the absolute number of the vocabulary within each sentiment category. The negative sentiment group features the highest count of unique vocabulary at 4558 words, indicating a broader lexical diversity. The positive sentiment follows with 3651 unique words, while the neutral sentiment shows the smallest vocabulary size with 3271 unique terms. This pattern reflects differences in how language is employed depending on sentiment, where negative reviews tend to include a wider variety of words possibly to cover a range of topics or nuances. Meanwhile, the positive and neutral reviews demonstrate a more limited range of word usage, which might indicate a more focused or consistent choice of expressions. The distinction in vocabulary size underscores how sentiment can influence not only the emotional tone but also the complexity and variety of language used.

**Fig. 5:**
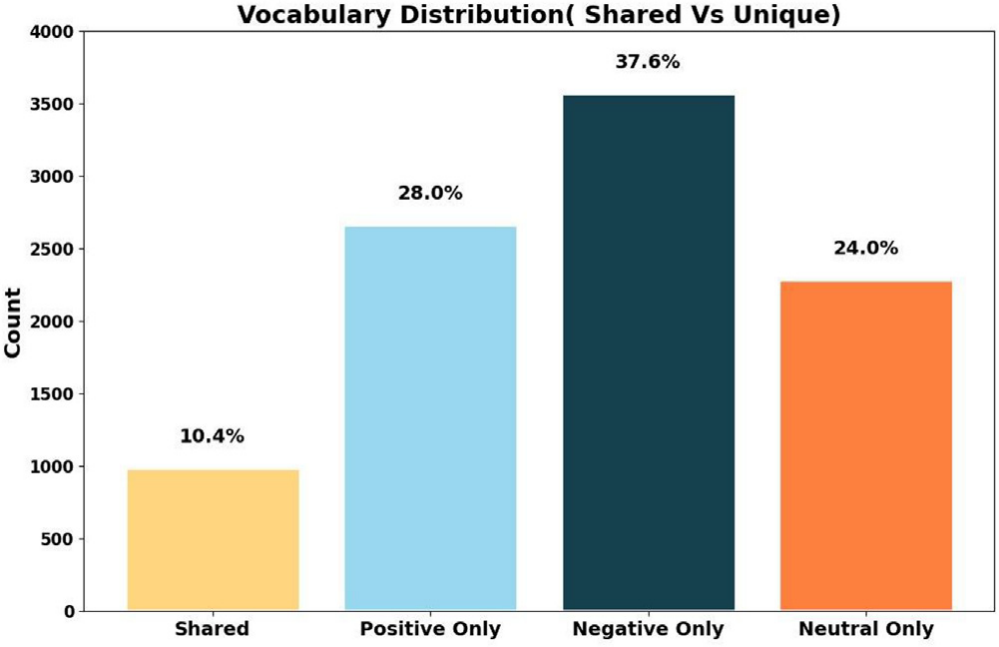
Shared vs unique vocabulary distribution among classes.

Fig. 5 presents the vocabulary distribution across sentiment classes. Out of a total vocabulary size of **9506** words, **987 (10.4** **%)** words are shared among all three classes. The remaining vocabulary is uniquely distributed, with **2664 (28.0** **%)** words exclusive to the positive class, **3571 (37.6** **%)** unique to the negative class, and **2284 (24.0** **%)** specific to the neutral class (Table 3, Table 4, Table 5, Table 6).

**Table 3:** Most common 30 words with their frequency.

**Table 4:** English translation of the most common 30 words with their frequency.

Positive		Negative		Neutral	
Words	Frequency	Words	Frequency	Words	Frequency
And	465	And	1029	However	915
Does	431	Does	957	Not	468
This	410	Was	768	But	378
Very	329	A / An	407	Good	348
It	305	Which	330	Can	333
Many / Much	267	Goes	329	Some	313
For	264	To do	320	Somewhat	270
Very	255	Is	297	Does	265
My	247	As a result	246	Doing	264
Good	218	No / Not	227	For	249
I	214	Very	217	It	237
Goes	206	Whose	209	To be	217
To do	196	For	207	Is / Exists	216
Use	183	My	196	Goes	215
No / Not	148	Lack	195	No / Not	194
A / An	145	Becomes	195	Use	159
Of	131	It	188	More	152
Good	129	Difficult	161	Correct / Exactly	148
With	127	Time	151	Very	139
New	125	With	146	Happens / Is	136
Beautiful	124	Dense	143	Work	135
Extremely	122	Raises	140	Fairly	130
Easy	120	Gives	136	Standard / Quality	118
Work	119	Not	132	Less	103
And	117	Create	126	Has been	100
Happens / Is	113	Or	114	Think / Feel	99
Comfortable	107	Doing	113	Was	98
Each	100	Smell	109	A little	97
Many / Much	267	A / An	407	And	92

**Table 5:** Top 20 bigrams among all sentences with their frequency.

**Table 6:** Top 20 trigrams among all sentences with their frequency.

**Fig. 6:**
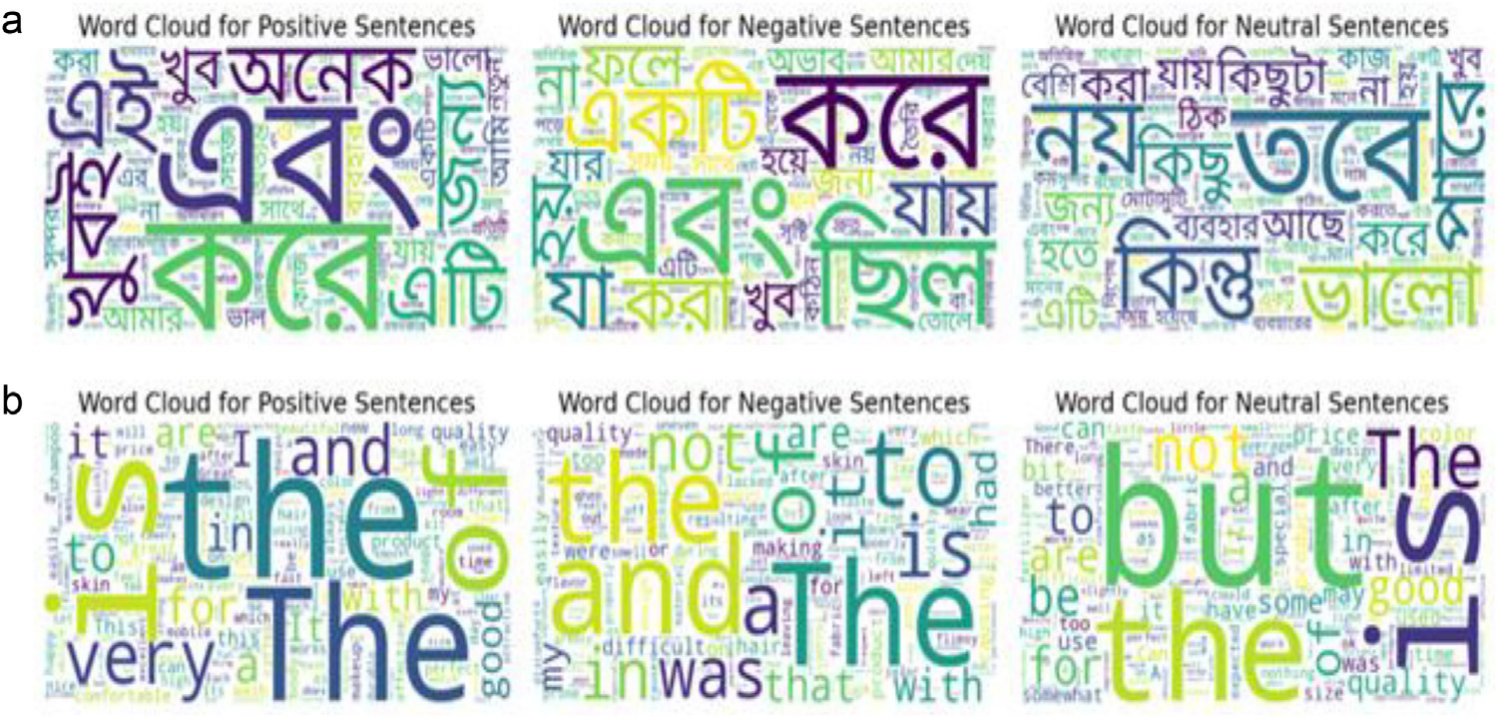
(a): Word clouds per class for Bangla forms. (b): Word clouds per class for English forms.

Fig. 6 (a) represents word clouds of Bangla sentences separately for positive, negative and neutral classes from the dataset, which graphically illustrates the frequency and prominence of terms inside their appropriate sentence form based on their appearance in the dataset. Fig. 6 (b) represents word clouds for English terms of these sentences respectively. However, the study contains stop words, which are popular words with little significance and are normally omitted from text analysis. Their inclusion may not provide a complete view of the dataset's most important keywords [3]. Despite this constraint, studying high-frequency terms gives useful information about the dataset's common lexicon and linguistic trends.

## 4 Experimental Design, Materials and Methods

**Fig. 7:**
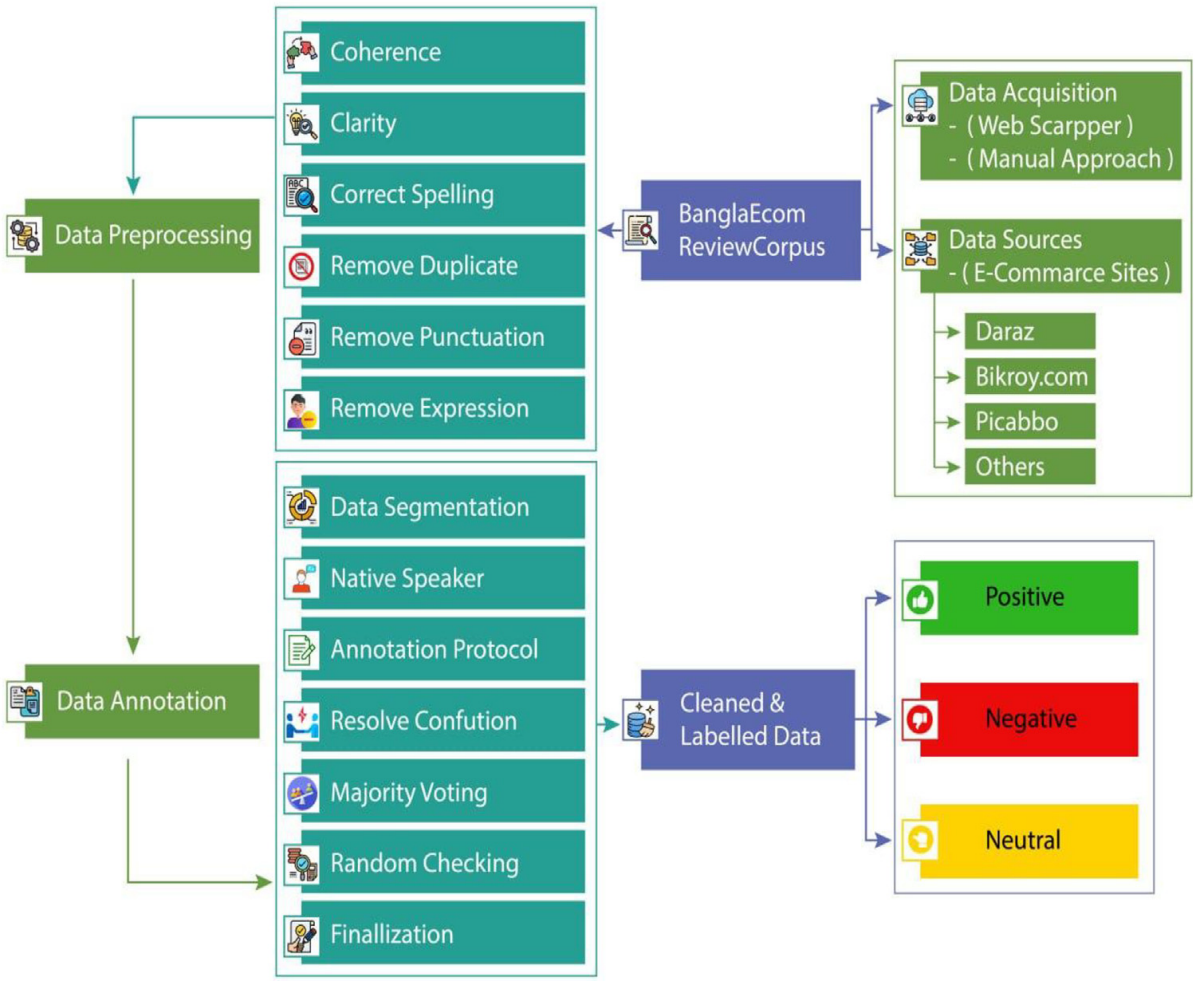
Data collection and preprocessing to create BanglaEcomReviewCorpus dataset.

The BanglaEcomReviewCorpus was developed through a systematic and ethically sound data acquisition process aimed at ensuring transparency, reproducibility, and high-quality data for Bangla sentiment analysis. The dataset was compiled between February and April 2024, focusing on customer reviews written in Bangla across major e-commerce platforms, including Daraz, Bikroy.com, and Pickaboo. A hybrid data acquisition technique was utilized, with a primary focus on automated web scraping. Using Python’s powerful BeautifulSoup library, pre-downloaded HTML files were systematically parsed to extract relevant review data based on precise CSS selectors. This automated web scraping method, applied on locally stored HTML content, enabled efficient and accurate data extraction without the need for live HTTP requests or handling dynamic web content. Where web scraping proved infeasible, manual review collection was employed to ensure comprehensive coverage of lingusitic diversity. Once collected, the dataset undergoes comprehensive preprocessing to enhance clarity and consistency. This stage includes ensuring coherence, correcting spelling errors, removing duplicates, eliminating punctuation, and filtering out unnecessary expressions to maintain data integrity. Following preprocessing, the data annotation phase begins, where native Bangla speakers meticulously label each sentence as **Positive, Negative, or Neutral** based on well-defined criteria. Annotators work independently to segment data, apply annotation protocols, and resolve ambiguities to ensure accuracy. To minimize bias and enhance reliability, disagreements in labeling are resolved through majority voting and random quality checks are conducted to verify consistency. Finally, the cleaned and labeled dataset is compiled and stored for future use in NLP research, AI model training, and business analytics. This structured methodology ensures that the BanglaEcomReviewCorpus is a high-quality, reliable dataset, contributing significantly to Bangla sentiment analysis and interdisciplinary research (Fig. 7).

After acquiring the raw Bangla text, the BanglaEcomReviewCorpus undergoes a structured preprocessing pipeline to ensure its quality and usability for sentiment analysis. The initial cleaning phase focuses on improving coherence, clarity, and word spelling, ensuring that the dataset maintains linguistic accuracy. Any identified errors are either manually corrected or removed to preserve data integrity. Following this, duplicate entries are detected and eliminated to prevent redundancy and ensure uniqueness in the dataset. Punctuation marks and unnecessary expressions are removed to simplify the text while maintaining readability. Additionally, regular expressions are applied to identify and remove unwanted patterns, further refining the dataset. Stop words, which do not contribute to sentiment analysis, are also excluded to enhance model performance. Once preprocessing is complete, the dataset moves into the annotation phase, where native Bangla speakers label the text as Positive, Negative, or Neutral based on predefined criteria.

To ensure annotation consistency and reliability, Inter-Annotator Agreement (IAA) is measured using Fleiss' Kappa, and any discrepancies in labeling are resolved through majority voting and expert review. Finally, random quality checks are performed before the cleaned and labeled dataset is compiled for further analysis and model training. This rigorous preprocessing and annotation workflow guarantees that the BanglaEcomReviewCorpus is a high-quality, well-structured resource for sentiment analysis in Bangla. Algorithm 1 depicts the data annotation process using Inter Annotator Agreement (IAA) [4]. Finally, we acquire cleaned and labelled data categorised as positive negative and neutral sentiments

**Algorithm 1:** BanglaEcomReviewCorpus data annotation process.

Input:	Raw Bangla Review, B_Sentence_
Output:	Sentiment of Bangla Review, BSentiment
Step-1:	Take all of the sentences for annotation
Step-2:	Select a group of native Bengali speakers (e.g., 4 annotators in 3 step) having strong language proficiency
Step-3:	Assign each sentences to multiple annotators (e.g., 3 annotators per sentence) to ensure redundancy
Step-4:	Use ‘Google Spreadsheet’ where annotators can label each sentence as saint, or common
Step-5:	Calculate agreement using statistical measures (i.e., Fleiss' Kappa) to calculate the inter-annotator agreement (IAA)
Step-6:	Calculate Fleiss’ Kappa:
Step-7:	$FK=\frac{\bar{A}-{\bar{A}}_{e}}{1-{\bar{A}}_{e}}\;\left\{ \begin{array}{c} Where,\;\bar{A}\;is\;the\;mean\;of\;the\;observed\;agreement,\;\\ {\bar{A}}_{e}\;is\;the\;mean\;of\;the\;expected\;agreement\;by\;chance \end{array}\right.$
Step-8:	Check conflict of resolution, sort them for further review and annotate with expert annotator
Step-9:	Measure majority voting for each form and set the final annotation with the highest vote
Step-10:	Calculate the confidence score for each sentence based on the proportion of annotators who agreed on the label
Step-11:	$Confidence\;Score=\frac{Number\;of\;AnnotatorsAgreeing}{Total\;Number\;of\;Annotators}\;\{\;A\;high\;confidence\;score\;indicates\;the\;high\;reliablity$
Step-12:	Check randomly samples annotated sentences and perform a quality check to ensure annotations meet the required standards
Step-13:	Compile the final annotated sentences into the BanglaEcomReviewCorpus dataset

BanglaEcomReviewCorpus is categorized into three distinct sentiment classes: Positive, Negative, and Neutral. These categories encompass a diverse range of opinions and sentiments provided by users.

**Positive Sentiment**: This category includes data where users provide favorable or satisfactory feedback. For example, one comment states,  (The lamp beautifully distributes the light in the room)”.

1. **Negative Sentiment**: This class contains feedback that expresses dissatisfaction or unfavorable opinions. For instance, one review reads,  (Quality is lower than expected)," illustrating the user's disappointment.
2. **Neutral Sentiment**: This category represents feedback that is neither explicitly positive nor negative. It may include ambiguous or mixed opinions, such as,  (The place is beautiful, but a bit warm)." This statement neither favors nor criticizes the product and reflects a neutral stance.

The BanglaEcomReviewCorpus study employed a systematic and meticulous data annotation process manually to analyse customer feedback on online products. Each review was categorized into **Positive, Negative, or Neutral** sentiments, which were numerically encoded as **Positive (2), Negative (0), and Neutral (1)** for computational processing. The annotation process was conducted by four expert annotators (two male and two female), selected based on their linguistic proficiency, academic qualifications, and familiarity with eCommerce terminology. Their expertise ensured accuracy, consistency, and cultural relevance in sentiment classification. To maintain uniformity and reduce bias, annotators worked independently with predefined criteria and clear examples. Neutral feedback was defined as balanced or ambiguous opinions, and specific guidelines were established to resolve uncertain cases.

A three-stage annotation strategy was implemented for Bengali ecommerce product review categorization:

1. **Sentence Allocation:** Each sentence was assigned to four native Bengali speakers for independent review.
2. **Classification & Agreement Check:** Annotators used Google Spreadsheets to label sentences as Positive, Negative, and Neutral, following rules. Statistical methods like Fleiss’ Kappa measured inter-annotator agreement (IAA).
3. **Conflict Resolution & Final Annotation:** Disputed labels were reviewed and reconciled by an expert annotator. Final classification was determined by majority voting, and a confidence score was calculated based on annotator agreement levels.

Additionally, random quality checks ensured that annotations met the required standards. The finalized dataset was compiled and stored in **``BanglaEcomReviewCorpus.xlsx''** for future analysis, establishing a highly reliable and well-structured corpus for sentiment analysis and NLP applications.

The accuracy of five deep learning models in categorizing text data into three sentiment classes—**Positive, Negative,** and **Neutral**—was evaluated using the **BanglaEcomReviewCorpus** dataset. Each model was trained separately for 50 epochs, with a batch size of 64, meaning the model adjusted its weights after processing every 64 samples. A comparative performance analysis was conducted to assess the LSTM, Bi-LSTM, Conv1D, Conv1D-LSTM, and Conv1D-Bi-LSTM models, as shown in Table 7. The Conv1D model achieved the highest accuracy of 92.05 %, followed by the Bi-LSTM model with 90.21 % accuracy. The Conv1D-Bi-LSTM model performed slightly lower, achieving 89.76 % accuracy, while the Conv1D-LSTM model and LSTM model attained 89.17 % and 88.09 % accuracy, respectively. Among individual class performance, the LSTM model demonstrated the highest precision (93.51 %) for the Negative class, while Conv1D-Bi-LSTM and Conv1D-LSTM models achieved better recall scores for Negative and Neutral classes. The Conv1D model showed a balanced performance across all three sentiment classes, making it the most effective model for this dataset.

**Table 7:** Performance evaluation of deep learning models using BanglaEcomReviewCorpus dataset.

Model	Class	Precision	Recall	F1 Score	Accuracy
**Conv1D-Bi-LSTM**	Negative	0.9054	0.9209	0.9131	0.8976
	Neutral	0.8854	0.8885	0.8869	
	Positive	0.9017	0.8831	0.8923	
**LSTM**	Negative	0.9351	0.8688	0.9007	0.8809
	Neutral	0.8267	0.9016	0.8625	
	Positive	0.9373	0.9088	0.9228	
**Conv1D**	Negative	0.9243	0.9244	0.9243	0.9205
	Neutral	0.9194	0.9161	0.9177	
	Positive	0.918	0.9211	0.9195	
**Bi-LSTM**	Negative	0.9421	0.8905	0.9156	0.9021
	Neutral	0.8591	0.9078	0.8828	
	Positive	0.9099	0.9071	0.9085	
**Conv1D-LSTM**	Negative	0.8742	0.9362	0.9041	0.8917
	Neutral	0.8583	0.8583	0.8583	
	Positive	0.9359	0.8795	0.9068	

## Limitations

The BanglaEcomReviewCorpus dataset, while valuable for sentiment analysis in Bangla e-commerce reviews, has certain limitations. One major limitation is that the dataset is specific to e-commerce reviews and may not be directly applicable to other domains like social media, news articles, or general opinion mining. Manual annotation of sentiment labels introduces the possibility of bias and inconsistency, as sentiment perception can vary among annotators. The dataset might not fully capture the linguistic diversity of Bangla, including regional dialects and informal expressions. The focus on structured sentiment classification may also overlook nuanced sentiments, sarcasm, and mixed opinions often present in user reviews. Despite these limitations, the BanglaEcomReviewCorpus dataset serves as a crucial resource for advancing sentiment analysis in Bangla e-commerce platforms

## Ethics Statement

The BanglaEcomReviewCorpus dataset was collected from publicly available e-commerce platforms, ensuring compliance with ethical guidelines for data acquisition. No personally identifiable information (PII) was included in the dataset, and all reviews were anonymized to protect user privacy. The dataset was processed and annotated following a structured protocol, including majority voting and random checking to minimize bias and ensure fairness. Ethical considerations were taken into account at every stage of the research to prevent any potential harm, including addressing biases in sentiment classification and ensuring that the dataset does not contain offensive or harmful content.

## CRediT Author Statement

**Umme Ayman:** Conceptualization, Data curation, Data Validation, Data Preprocessing, Statistical Analysis, Visualization, Software, Resources, Methodology design, Model Implementation, Writing – review & editing; **Tanvir Ahmded Akash:** Data Curation, Data Preprocessing, Data Validation, Data Statistical Analysis, Writing – review & editing; **Taslima Akter:** Data Curation, Data Preprocessing, Writing- Original Draft; **Saiham Zaman Mridul:** Data Preprocessing, Data Validation, Methodology diagram, Performance metrics calculation; **Yadad Sutradhar:** review and editing.

## Data Availability

Mendeley DataBanglaEcomReviewCorpus: A Resource for E-Commerce Product Review Sentiment Analysis (Original data).
